# Thyroid Hormones and Their Receptors: From Development to Disease

**DOI:** 10.4061/2011/284737

**Published:** 2012-04-09

**Authors:** Michelina Plateroti, Juan Bernal, Samuel Refetoff, Laurent Sachs

**Affiliations:** ^1^Centre de Génétique et de Physiologie Moléculaire et Cellulaire, Université Claude Bernard Lyon 1, 69622 Villeurbanne, France; ^2^Institut de Génomique Fonctionnelle de Lyon, Ecole Normale Supérieure de Lyon, 69364 Lyon, France; ^3^Instituto de Investigaciones Biomédicas, CSIC-UAM, Centro de Investigacion Biomedica en Enfermedades Raras (CIBERER), 28029 Madrid, Spain; ^4^Departments of Medicine, Pediatrics, and Genetics, University of Chicago, Chicago, IL 60637, USA; ^5^CNRS UMR7221, Evolution des Régulations Endocriniennes, Muséum National d'Histoire Naturelle, 75231 Paris, France

Thyroid hormone (TH) has a fundamental role in the development, growth, and metabolic homeostasis in all vertebrates by affecting the expression of a variety of genes. As a consequence, an altered thyroid status affects many organs and systems. TH action is mediated mainly by the TH receptors (TRs), which belong to the nuclear receptor superfamily of transcription factors. Given the rapidly accumulating data on the pleiotropic functions of the TH and TR, the aim of this special issue was to summarize the most recent advances in this field. In particular, investigators contributed with review articles that address the efforts to understand the function of the TH and TRs in development, growth, metabolism, and homeostasis. Moreover, reviews also focused on pathologies linked either to altered thyroid status or to gene alterations that modify hormone action.

We open this special issue with the paper by J. R. Tata, providing a historical perspective on the discovery of thyroid hormone, thyroid hormone receptors, and mechanisms of action. It also summarizes some of the current thinking on thyroid hormone action and proposes three models to explain the multiplicity of the thyroid hormone effects. Moreover, the paper highlights that the proposed models are not the ultimate mechanism of the action of thyroid hormone and that future studies will probably lead to the discovery of some fundamental principles of biological regulation and signalling as was done in the past. The paper by V. M. Darras et al. summarizes data available on thyroid hormone receptors in chicken and zebrafish, two major model systems for developmental biology. This contribution is of particular interest in reviewing the implication of TRs during development in nonmammalian species.

Two papers deal with the role of TH and TRs in the nervous system as they have addressed several aspects of TH action in the brain. Of particular interest is the regulation of T3 entry into neural cells. Brain T3 has dual sources, the circulation and local generation through T4 deiodination. The paper by P. Mohácsik et al. deals with mechanisms that regulate the synthesis of the nuclear receptor ligand T3 in the brain by the glial cells astrocytes and tanycytes as well as the cellular transport of T3 by specific membrane transporters. The paper by F. Chatonnet et al. is based on the work on cerebellar development carried out by the group of Frederic Flamant in Lyon. They address the multiple aspects of TH action in the brain, including the control of ligand production, the role of receptors, the regulated genes, the interactions among neural cells in TH actions, and the disruptions due to environmental contaminants.

Other papers focus on TH-alteration-dependent pathologies. TH acts in most body's tissues in a pleiotropic fashion. Two papers have addressed this issue focusing on several systems. The paper by M. Franco et al. is a general overview of the pleiotropic effects of TH on many different pathways and organ targets, based on studies on hypothyroidism. They review extensively the effect of hypothyroidism on mitochondria, ischemic reperfusion damage of the heart, renal physiology, vascular relaxation, and lipoprotein metabolism. The paper by M. Hage et al. reviews the actions of TH on carbohydrate metabolism with emphasis on the relationship between diabetes and thyroid disorders. Finally, paper by C. Pantos et al. is a careful review of the pathophysiological mechanisms underlying the reactivation of a foetal phenotype in the damaged myocardium and the eventual implication/significance of a TH-based therapy.

We close our special issues with the paper by M. D. Rosen and M. L. Privalsky dealing with TR mutations in cancer. This review starts with a very elegant historical perspective and the authors describe the TR signalling and the effect of TR mutations in endocrine diseases.

Taken together, the contributors of this special issue clearly underscore the complexity of the TH action from a cellular and molecular point of view. As suggested by Professor J. R. Tata's conclusive remarks, they also point out that much has yet to be learned about how TH signalling regulates specific gene expression and diverse cellular functions from early development to cell death in both physiological and pathological situations.

*Michelina Plateroti* *Michelina Plateroti* *Juan Bernal* *Juan Bernal* *Samuel Refetoff* *Samuel Refetoff* *Laurent Sachs* *Laurent Sachs*

